# Prediction of Birth Type Based on the Health Belief Model 

**Published:** 2017-09

**Authors:** Sakineh Dadipoor, Mitra Mehraban, Teamur Aghamolaei, Ali Ramezankhani, Ali Safari-Moradabadi

**Affiliations:** 1 Department of Public Health, Faculty of Health, Hormozgan University of Medical Sciences, Bandar Abbas, Iran; 2 Department of Obstetrics and Gynecology, Fertility and Infertility Research Center, Faculty of Medicine, Hormozgan University of Medical Sciences, Bandar Abbas, Iran; 3 Department of Public Health, School of Public Health, Shahid Beheshti University of Medical Sciences, Tehran, Iran; 4 Social Development & Health Promotion Research Center, School of Public Health, Kermanshah University of Medical Sciences, Kermanshah, Iran; Department of Public Health, School of Public Health, Shahid Beheshti University of Medical Sciences, Tehran, Iran

**Keywords:** Childbirth, C-Section, Primiparous Women, Health Belief Model

## Abstract

**Objective:** To anticipate the type of childbirth according to the health belief model.

**Materials and methods:** The present cross-sectional research was conducted on 222 primiparous women visiting the healthcare center in Khorram Abad. A combination of simple randomization and clustering was used to do the sampling. The data collection instrument was a validated four-part questionnaire the first part of which contained demographic information. The second part was comprised of awareness questions while the third dealt with the constructs of the health belief model. The final part consisted of the behavioral intention derived from the logical action theory. SPSS 16 was used to statistically analyze the data and the significance level was set at p ˂ 0.05.

**Results:** The average age of the participants was 27.40 ± 6.07 years. Intention to go for a vaginal birth showed to be significantly correlated with awareness, perceived sensitivity, intensity, barriers and benefits (p ˂ 0.001) as well as self-efficacy (p = 0.025). The best predictor of the type of childbirth turned out to be the perceived barriers (OR = 1.153, p ˂ 0.001) and only then awareness (OR = 1.108, p ˂ 0.001).

**Conclusion:** Strategies to remove the barriers of preferring vaginal childbirth, raising women’s awareness of the side effects of C-section and the benefits of vaginal birth, strategies to enhance women’s beliefs in their capability of natural childbirth can be used to reduce the prevalence of unnecessary C-sections.

## Introduction

Childbirth as a science which terminates pregnancy aims to maximize the chances of giving birth to a healthy infant without threatening mother’s health (1). In the majority of cases, vaginal birth is the best and the most [referred whereas a C-section is supposed to be limited to cases unable to have a natural delivery or when mother’s or infant’s health are threatened (2). According to the body of related literature, the side effects of a C-section are significantly greater than vaginal delivery (3). However, surgical childbirth has been reported as the most prevalent surgery on a global scale and its prevalence is ever increasing (4).

According to the existing statistics, C-section accounts for 25.7% of all deliveries and this rate is reported to be 2.3% in Angola and 46.2% in China (5). According to some a review research on 137 countries in 2010, the rate of C-section was below 10% in 54 countries, above 15% in 69 countries and between 10-15% in 41. Iran ranked second with 41.9% standing only next to Brazil with 45.9% of preference for a C-section. This rate is tremendously above the standard defined by the World health Organization (WHO) (6). This astronomic figure shows we truly need to find the underlying reason for such a high rate and think of ways to cut down on it and further approach the standard set by WHO. As childbirth has been described particularly to primiparous women as a horrifying experience, they need to be motivated for vaginal delivery and need to become more aware of the hazards of a C-section (7). The first step to predict and plan for health-related behaviors is to choose the right model. One such model is the health belief model in education (8). This model is based on the principle people show the right behavior once they feel in danger (perceived sensitivity construct); the hazards of the unhealthy behavior are intense (perceived intensity); the healthy behavior has got benefits for them (the perceived benefits); the barriers to the healthy behavior can be removed (perceived barriers); one is capable of showing the right behavior (perceived self-efficacy) (9). 

A vast majority of the related literature so far has only dealt with the prevalence of C-section and its correlates. Little work has focused on theory-driven and psychological factors. Due to the contrary results obtained based on the health belief model about the choice of childbirth type (10-12), Therefore, the present study aimed to explore the predictors of the type of childbirth based on this model.

## Materials and methods

The present descriptive, analytical study was cross-sectional in design. The target population was all primiparous women in their third month of pregnancy who visited the healthcare centers of Khorramabad. The sample size was determined to be 220 through a formula considering a correlation coefficient of 0.25 and CI of 0.95. The sampling method was a combination of simple randomization and clustering method. Initially, from among the existing 20 healthcare centers in Khorramabad, 12 were randomly selected. Then, pregnant visitors’ files were analyzed to extract qualified cases to enter the study. The inclusion criteria were: age range of 18-35 years, height above 145 centimeter, weight range of 50-90 kilos, primiparity, pregnancy age of at least 28 weeks and willingness to take part in the research. The exclusion criteria were: placenta problems, multiple pregnancy, narrow-shaped pelvis, pregnancy diabetes, history of hypertension, pregnancy preeclampsia and previous abortion. The data collection instrument was a reliable and valid questionnaire by Sharifi rad et al. (2009) (13). The target questionnaire was comprised of four sections the first of which dealt with demographic information (mother’s personal/social and midwifery information in 14 items). The second part was comprised of awareness questions (20 items: 15 four-choice and 5 three-choice). This section would be scored between 0 and 20. The third section dealt with the constituent constructs of the health belief model which was to be rated on a 5-level Likert scale. The constructs included: perceived sensitivity (6 items) (score: 6-30), perceived intensity (7 items) (score: 7-35), perceived barriers (17 items) (score: 17-85), perceived benefits (21 items) (score: 21-105) and perceived self-efficacy (5 items) (score 5-25). The fourth section of the questionnaire was concerned with behavioral intention derived from the logical action theory (1 item: either vaginal or surgical). Once the required permission was obtained from the university deputy of research, the data were collected. All particpants were ensured of the confidentiality of the data and fully consented to take part. The collected data entered SPSS 16 for the required statistical analyses. Mean scores and standard deviation were used to describe the data. Further analyses including independent-sample t-test, chi-squared test and logistic regression were run too. The level of significance was set at p ˂ 0.05.

## Results

The mean age of the participants was 27.40 ± 6.07 years; their mean age of marriage was 23.40 ± 5.20 years. 17.7% of the participants were below 20 years of age; 79.5% had a moderate socioeconomic level’ 74.1% of them were housewives and finally 49.1% held a diploma (Table 1).

**Table 1:** Distribution of participants’ demographic information

**Variable**	**Group**	**Frequency**	**Percentage**
Age	˂ 20	39	17.7
	20-35	148	67.3
	˃ 35	33	15
Socioeconomic level	Moderate	175	79.5
	Above moderate	45	20.5
Education	˂ Diploma	79	35.9
	Diploma	108	49.1
	University degree	33	15
Employment	Unemployed	163	74.1
	Employed	57	25.9

As the findings revealed, statistically significant correlations were found between birth type and: awareness (p ˂ 0.001), perceived sensitivity (p ˂ 0.001), perceived intensity (p ˂ 0.001), perceived barriers (p ˂ 0.001), perceived benefits (p ˂ 0.001) and self-efficacy (p = 0.025).

Across all constructs the significant findings belonged to those who opted for a vaginal birth (Table 2). Moreover, the results revealed that 93 participants (66.4%) intended to go for a vaginal delivery while 47 (33.6%) chose C-section. 

Logistic regression analysis was conducted for the predictors of fertility-related factors and the type of childbirth. The independent variables awareness, perceived sensitivity, barriers, benefits, intensity and self-efficacy entered the model. The most determining predictor of the type of birth was perceived barriers (OR=1.153) (p˂.001) and only then awareness (OR = 1.108) (p ˂ 0.001) (Table 3).

## Discussion

The present study sought to determine the predictors of the birth type among primiparous women in Khorramabad according to the health belief model. According to the present findings, the number of those who intended to have a vaginal delivery was twice as many as those who went for a C-section. These findings were consistent with those reported by Yuen Loke et al. (11) and Yousef zadeh et al. (14). However, a lower percentage was reported in the Iranian body of literature than Asia (96.9%), Singapore (95.1%) and Turkey (84.1%) (15-17). These contradictions could be due to the social and demographic features of the women participants or cultural differences and healthcare services provided in different countries. Anderson indicated that the culture in many communities is involved in physician’s decision whether to perform a C-section or not (18). 

In the present research, about one-third of the participants tended to have a C-section which was higher than that in Yuen Loke et al. (11). This finding shows that a great many women in Khorramabad preferred a surgical childbirth which can be due to many factors including the cultural, social and demographic. Therefore, there is a need for further investigations of the factors involved in the increasing rate of C-section. 

**Table 2:** Distribution of mean and standard deviation of awareness and health belief model constructs

**Model construct**	**Type of birth**		**Significance level** *
	**Vaginal**	**Surgical**	
	**Mean ± SD**	**Mean ± SD**	
Awareness	48.58 ± 11.20	35.34 ± 12.11	˂ 0.001
Perceived sensitivity	48.75 ± 11.62	41.37 ± 12.69	˂ 0.001
Perceived intensity	49.02 ± 10.08	44.02 ± 9.01	˂ 0.001
Perceived barriers	48.85 ± 9.66	37.80 ± 8.41	˂ 0.001
Perceived benefits	56.78 ± 9.67	49.91 ± 8.07	˂ 0.001
Perceived self-efficacy	33.98 ± 14.95	28.02 ± 12.55	0.025

* T-test

**Table 3:** Predictors of behavioral intention of the birth type based on the health belief model

**Variables**	**B (beta)**	**p-value** *	**Exp (B)**	**CI 95%**	
				**Low**	**High**
Awareness	0.103	0.000	1.108	1.052	1.167
Perceived sensitivity	-0.018	0.459	0.982	0.936	1.030
Perceived intensity	-0.009	0.788	0.991	0.925	1.061
Perceived barriers	0.142	0.000	1.153	1.084	1.225
Perceived benefits	-0.049	0.087	1.050	0.993	1.110
Perceived self-efficacy	0.006	0.742	1.006	0.969	1.045

* logistic regression

The key predictor of the type of childbirth in the present research was found to be awareness and perceived barriers. Sharifi rad et al. indicated that perceived barriers formed the primary construct of the health belief model in suggesting a healthy behavior (19). The fear of natural delivery was reported by Howharn et al. and Negahban et al. (20) as the key barrier of vaginal childbirth (21) In Nerum research, consultation managed to change the decision of 93% of those fearing natural delivery  (22). Therefore, attention to the psychological aspect of interventions and following strategies to remove the perceived barriers to natural delivery can be truly helpful. In the present research, the key predictor of birth type showed to be the perceived barriers. Awareness was among the kay factors involved in health and awareness arising was the first step of making a health-related decision. The role awareness plays in choosing a C-section is determining (23). In their research, Anderson et al. concluded that in order to reduce the prevalence of C-section and its side effects, pregnant women’s awareness should be raised (18). Biglarifard’s investigation revealed that raising mothers’ awareness natural delivery led to a more positive attitude towards this type of childbirth. it can, therefore, be expected that higher awareness and consequently developing a correct attitude towards natural childbirth can significantly affect mother’s decision to choose the type of delivery (2). 

The results revealed a statistically significant correlation between perceived sensitivity and the intention of natural childbirth. In other words, the mean score of perceived sensitivity was higher in women who intended to have vaginal delivery. Fuglenes et al. (24) drew attention to perceived sensitivity as a factor involved in selecting the type of childbirth. It appears that women’s higher awareness of the side effects of a C-section can increase their perceived sensitivity since these constructed are linearly related.

According to the present findings, the perceived intensity and one’s intention of natural delivery showed to be significantly correlated. As pinpointed by Disney et al. (25), the perceived risk of each delivery type for both mother and child play a role in selecting the type of delivery. A body of related research confirmed the effect of perceived intensity on the type of childbirth (26, 27) while others including Baghianimoghaddam did not (28). This divergence in findings can be due to the fact that mothers, if made adequately aware of the benefits of natural delivery, can more prevalently show this behavior than if provided with the side effects of a C-section. 

Moreover, as the present findings revealed, perceived benefits showed to be significantly correlated with the birth type. In fact, the mean score of perceived benefits was high in women who intended to have a natural delivery. These findings were consistent with those by Yuen Loke et al. (11), and Hassani et al. (29). This further shows that accepting the benefits of natural delivery and overcoming the existing barriers are essential to reduce the rate of unessential cesareans. 

The present findings further revealed that self-efficacy and birth-type were significantly correlated. In other words, the mean self-efficacy score was higher in women who intended to have a natural birth. Kan’ani et al. indicated that self-efficacy was effective in the choice of the birth type (30). Dilk et al. reported self-efficacy as a key factor involved in selecting the right type of childbirth especially the natural (31). Khorsandi et al. emphasized on self-efficacy as the key factor involved in birth type and controlling the fear of natural birth (32). Self-efficacy dramatically influences one’s motivation and further encourages one to opt for natural delivery. Therefore, special attention should be paid to perceived self-efficacy in the instructions provided to prospective mothers and appropriate strategies should be followed to enhance their belief in capability of handling a natural birth and lowering the pain involved. 

One limitation of the present research was that all women visitors were urban residents and there was no access to rural residents. The limited sample size, the mere incorporation of personal factors involved in selecting the birth type and neglecting social and contextual factors are among the other limitations.

## Conclusion

Considering the predictors of the birth type, there seems to be a great need for strategies to remove the barriers to natural delivery. These can include a correct way of raising mothers’ awareness and changing their wrong perceptions of natural delivery and its pain. These can be accompanied by efforts to correct women’s belief in their capability of handling a natural birth. All these can help to cut down on the rate of unnecessary surgical deliveries.
